# Prevalence and Associated Features of Anxiety Disorder Comorbidity in Bipolar Disorder: A Meta-Analysis and Meta-Regression Study

**DOI:** 10.3389/fpsyt.2018.00229

**Published:** 2018-06-27

**Authors:** Hale Yapici Eser, Anil S. Kacar, Can M. Kilciksiz, Merve Yalçinay-Inan, Dost Ongur

**Affiliations:** ^1^School of Medicine, Koç University, Sariyer, Turkey; ^2^Research Center for Translational Medicine, Koç University, Istanbul, Turkey; ^3^Psychotic Disorders Division, McLean Hospital, Belmont, CA, United States; ^4^Department of Psychiatry, Harvard Medical School, Boston, MA, United States; ^5^Neurological Sciences, Koç University Hospital, Istanbul, Turkey

**Keywords:** bipolar disorder, anxiety disorders, epidemiology, panic disorder, psychosis, substance-related disorders

## Abstract

**Objective:** Bipolar disorder is highly comorbid with anxiety disorders, however current and lifetime comorbidity patterns of each anxiety disorder and their associated features are not well studied. Here, we aimed to conduct a meta-analysis and meta-regression study of current evidence.

**Method:** We searched PubMed to access relevant articles published until September 2015, using the keywords “Bipolar disorder” or “Affective Psychosis” or “manic depressive” separately with “generalized anxiety,” “panic disorder,” “social phobia,” “obsessive compulsive,” and “anxiety.” Variables for associated features and prevalence of anxiety disorders were carefully extracted.

**Results:** Lifetime any anxiety disorder comorbidity in BD was 40.5%; panic disorder (PD) 18.1%, generalized anxiety disorder (GAD) 13.3%, social anxiety disorder (SAD) 13.5% and obsessive compulsive disorder (OCD) 9.7%. Current any anxiety disorder comorbidity in BD is 38.2%; GAD is 15.2%, PD 13.3%, SAD 11.7%, and OCD 9.9%. When studies reporting data about comorbidities in BDI or BDII were analyzed separately, lifetime any anxiety disorder comorbidity in BDI and BDII were 38% and 34%, PD was 15% and 15%, GAD was 14% and 16.6%, SAD was 8% and 13%, OCD was 8% and 10%, respectively. Current any DSM anxiety disorder comorbidity in BDI or BDII were 31% and 37%, PD was 9% and 13%, GAD was 8% and 12%, SAD was 7% and 11%, and OCD was 8% and 7%, respectively. The percentage of manic patients and age of onset of BD tended to have a significant impact on anxiety disorders. Percentage of BD I patients significantly decreased the prevalence of panic disorder and social anxiety disorder. A higher rate of substance use disorder was associated with greater BD–SAD comorbidity. History of psychotic features significantly affected current PD and GAD.

**Conclusions:** Anxiety disorder comorbidity is high in BD with somewhat lower rates in BDI vs BDII. Age of onset, substance use disorders, and percentage of patients in a manic episode or with psychotic features influences anxiety disorder comorbidity.

## Introduction

With an average 1% lifetime prevalence for bipolar disorder type I (BD I) and overall prevalence of almost 4% for bipolar spectrum disorders more broadly (1), BD is one of the most prevalent psychiatric disorders. On the other hand, it is also one of the most mutable disorders, comorbidity is the norm among patients diagnosed with BD and this may complicate the differential diagnosis as well as management (2–5). Given these challenges, it is important to understand the patterns of comorbidity and complex presentations in patients with BD.

Among BD comorbidities, anxiety disorders are second only to substance use disorders (SUD) (6–8), and in some reports even more common (9, 10). Only one study in a Chinese population found low anxiety disorder rates in BD patients (11). In addition, BD patients have higher rates of comorbid anxiety disorders compared to general population (2, 12, 13) and compared to unipolar depression patients (14, 15). The comorbidity of anxiety disorders in BD is not only common but also clinically significant because it can affect patient course and treatment response (16–21). Individual studies found an association of anxiety disorder comorbidity with earlier age of onset (22–24), increased symptom severity (17, 23), and multiple adverse outcomes in BD (17–19, 21, 23–47). BD patients diagnosed with anxiety may also need to be managed differently (48, 49). At a deeper level, it is not known whether the BD-anxiety disorder comorbidity represents a shared pathophysiology between two common psychiatric conditions, or a subgroup of patients with a genuinely different clinical condition which appears comorbidity. Ultimately, BD may come to be seen not only as a mood disorder but also as complex presentation with many nested circles involving anxiety, substance use, character pathology, and physical conditions (50).

Although there is a literature on the topic, the moderators of BD-anxiety disorder comorbidity and the variance in subgroups of anxiety disorders are not well studied due to the heterogeneity of BD and the heterogeneity in study methodology. Individual studies point out that unipolar mania patients may be less likely to be diagnosed with an anxiety disorder (51), and that BD type II (BD II) is found to be more related with anxiety symptoms (13, 36, 52) and it can be comparable to patients diagnosed with major depressive disorder (MDD) (14, 15, 52).

There is also the question of whether specific anxiety disorders are observed more often than others in BD patients, and which variables are important for comorbidity with each anxiety disorder. For example, association of specific anxiety disorders with gender, age at onset of BD, and substance use disorder may offer clues about the patterns of disorder emergence (10, 53–57). Finally, this research may point to risk factors such as traumatic life events or shared genetic vulnerability (53–56).

In this study, we conducted a systematic review and meta-analysis to answer the following questions about BD and anxiety disorder comorbidity: Question 1: What is the prevalence of lifetime and current anxiety disorder diagnosis in BD? We broke this question further to examine comorbidity patterns of commonly reported disorders: generalized anxiety disorder, panic disorder, obsessive compulsive disorder and social phobia. We also examined data from BD overall, as well as specifically in BD I and BD II. Question 2: How do moderators such as the mean age, marital status, gender, BD I ratio, educational level, age of onset of BD, current episode of BD, and presence of substance use disorders affect anxiety disorder comorbidity in BD? We sought to evaluate a large literature and identify patterns which may help provide new insights into the pathophysiology and clinical course of these common disorders.

## Methods

### Search strategy

**Figure 1:**
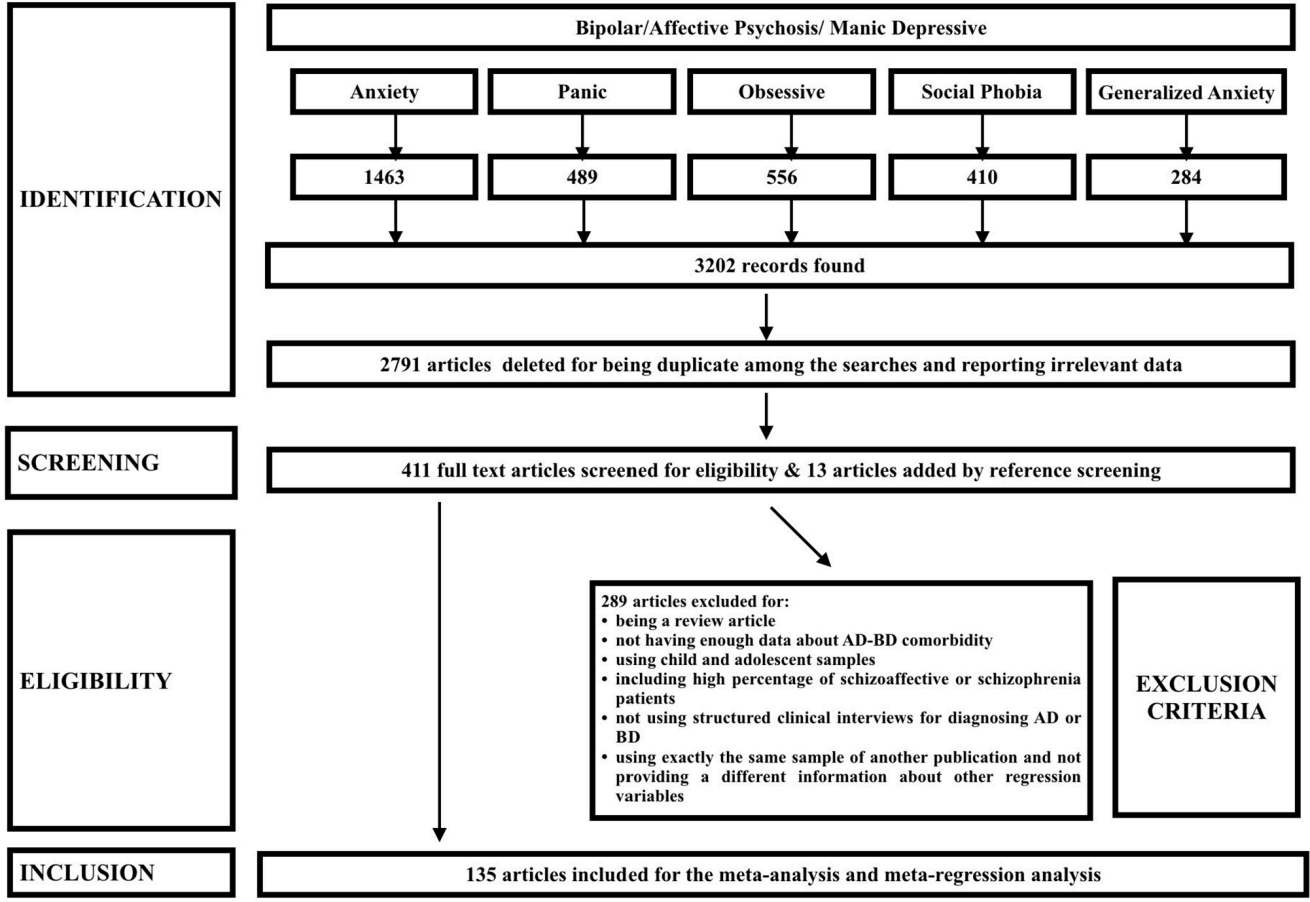
PRISMA flow of found studies based on the research and screening methods.

This study was designed and conducted in accordance with the MOOSE (58) and PRISMA (59) guidelines. We searched PubMed to access all relevant articles published until September 30th, 2015, starting from the first published viewable study in 1995. We used the keywords “Bipolar disorder” or “Affective Psychosis” or “manic depressive” separately with “generalized anxiety,” “panic disorder,” “social phobia,” “obsessive compulsive,” and “anxiety” to search for all relevant articles with this topic. All keywords were searched in the “Title/Abstract” of the articles. Only the articles that had an abstract or full text in published in English were included in the study (Figure 1).

All abstracts were read to identify articles that have relevant prevalence data of any anxiety disorder in BD. If the abstract was inconclusive, the full article was accessed to ascertain whether relevant information was reported. Articles that reported data about prevalence of anxiety disorders were saved in full text for further evaluation. References of all original articles and reviews were checked for additional studies not previously accessed.

### Study selection

Only original articles that diagnosed BD based on structured interviews [SCID, SADS- Schedule for Affective Disorders for Schizophrenia, DIGS (Diagnostic interview for genetic studies), CIDI or MINI] or on selected diagnostic criteria (both DSM-III and DSM-IV) were included. Studies that did not present conclusive data about BD—anxiety disorder comorbidity, review articles, studies on child and adolescent bipolar disorder patient samples, studies that were performed on data from health records/registries or hospital charts, studies that did not use structured clinical interviews for the diagnosis of anxiety disorders and diagnosed anxiety disorders based on scales, studies that reported data about the prevalence of “any anxiety disorder diagnosis” but did not define or clarify which disorders were concluded as “any anxiety disorder” were excluded.

Studies that presented data of comorbidity in a heterogenous sample of psychotic disorders (i.e., schizophrenia, schizoaffective disorder and bipolar disorder) were excluded, however, studies that included less than 5% of patients schizoaffective disorder in addition to BD were included because of potential overlap in DSM-III criteria. Samples consisting of one gender or samples with another disorder comorbidity in the whole sample (e.g., eating disorder or substance use disorder) were also included for the pooled analysis. Data about past history of intraepisode anxiety disorder were excluded (60, 61).

### Data extraction

All relevant data about the prevalence and possible determinants of anxiety disorder comorbidity were extracted from each article by two authors (HYE, CMK, ASK, MY); HYE and ASK subsequently checked for the correctness of each data from each article. Extracted information included: name of the authors, name of the city and country where the sample was recruited, publication year, purpose of the study, how the research sample was defined and method of sampling, types of bipolarity in the sample (BD, Bipolar I, II) and percentage of BD I in the sample, sample size, number of individuals diagnosed with any anxiety disorder, generalized anxiety disorder, panic disorder, social phobia, and obsessive compulsive disorder in BD, BD I, BD II separately, mean age of the sample, age range, gender ratio, interview method, how bipolar disorder is diagnosed, if psychotic features are reported or not and percentage of patients with a history of psychotic symptoms, how anxiety disorder is diagnosed, if anxiety diagnosis is current, lifetime, last year or last month, definition for “any anxiety disorder,” episode of BD, BD age of onset, % of high school or higher graduates, marital status of the sample, BD family history, anxiety disorder family history, ADHD prevalence in the sample, and substance use disorder prevalence in the sample.

When a study did not report if the anxiety disorder diagnosis was current or lifetime, studies that used the MINI were coded as “current diagnosis”; “Diagnostic Interview for Genetic Studies” were coded as “lifetime.” A SCID diagnosis of anxiety disorders were coded as “lifetime” unless otherwise specified. A CIDI diagnosis of GAD was coded as last 12 months and defined as current, whereas a panic disorder diagnosis was coded as “lifetime,” unless reported otherwise except for Perich et al. (62), where diagnosis was stated as comorbid. Comorbidity reported as based on DSM-IV criteria was grouped in current diagnosis (44).

Studies were included to the analysis regardless of inpatient-outpatient populations or current episode of BD or BD type. However, % of patients in a manic episode were coded for use as a covariable in the meta-regression analysis.

Studies from the same clinics and research groups were examined for overlapping data and differences in reporting. In cases where two studies were performed on the same dataset (for example, Stanley Foundation, Step-BD, NESARC or JORVI study samples), the study with the most up to date data and the largest sample size was used in the analysis. Two authors agreed to use which data should be used in the study. In cases where meta-regression variable was presented in one article overlapping with a more up to date study, this variable and the prevalence for that study were only included in the meta-regression analysis of that variable, but not in the overall prevalence analysis.

Only studies that included all DSM-IV anxiety disorders in “any anxiety disorder” classification were coded for this variable. In cases where the prevalence of each anxiety disorder was reported separately, data were not summed for “any anxiety disorder” considering the possibility of comorbidity of anxiety disorders. If a study did not report which anxiety disorders they included in the any anxiety disorder definition but stated diagnosing anxiety disorder based on DSM-IV, it was assumed that data presented the overall prevalence of all anxiety disorders. Prevalence of substance use disorder comorbidity in the sample included both alcohol and substance use disorders, and due to lack of definition both current and lifetime prevalence data of SUD were pooled together in the meta-analysis.

### Statistical analyses

Meta-analysis and meta-regression analysis were conducted using the Comprehensive Meta-analysis software licensed to Koc University. First, a publication bias analysis was conducted by visualization of the Funnel plots, Egger's test (63) and the Begg-Mazumdar Kendall's tau (64). Heterogeneity was assessed with the I2 and Q statistic for each analysis. Secondly, we conducted a meta-analysis using a random effects model for all the studies that included data about an anxiety disorder diagnosis in all types of BD. Data were analyzed separately for lifetime and current comorbidity. Second, we conducted a meta-analysis on BD I and BD II subgroups.

The meta-regression analyses were conducted for measuring the effect of possible variables on the heterogeneity of included studies. These variables included % of BD I patients in the sample, mean age of the sample, % of patients married, % of patients in a manic episode during the assessment, % of patients with a history of psychosis, % with high school or higher education, % of females in the sample, duration of BD, age of BD onset, % of patients diagnosed with ADHD, % of patients with a substance use disorder and family history of BD. For both lifetime and current comorbidity analyses, we conducted a univariate regression with random-effects model using mixed effect regression (unrestricted maximum likelihood). We could not analyze the effect of family history of anxiety disorders due to lack of adequate number of studies.

## Results

### Description of the included studies

After identification, screening and eligibility assessments, data from 152 studies published between 1995 and 2015 were included in the study and data from 135 articles were used for meta-analysis and meta-regression studies after excluding studies with overlapping samples. Figure 1 shows the PRISMA flow of selected studies. Sample sizes ranged from 20 to 3,766. Data from 121 studies were used for prevalence and data from 130 studies were used for meta-regression calculations. Number of studies and total sample sizes of each variable are presented in Tables 1–**4**.

**Table 1:** Lifetime comorbidity of anxiety disorders in bipolar disorder.

**Lifetime Comorbidity**	**No of studies**	**No of Patients**	**Prevalence % (CI)**	**Heterogeneity I2**	**Cochran Q (*p-*value)**	**References**
Any anxiety disorder	39	13,409	40.5 (36.0–45.2)	96.09	971.7 (*p* < 0.0001)	(13, 16, 17, 19, 22, 30, 34, 39, 40, 42, 46, 47, 51, 55, 56, 76, 89–111)
Panic disorder	63	14,890	18.1 (15.8–20.5)	93.2	906.6 (*p* < 0.0001)	(2, 9, 11, 13, 15–19, 22, 30, 32, 34, 38, 42, 46, 47, 52, 57, 76, 77, 83–85, 89–92, 95–100, 102, 103, 106, 107, 109, 112–136)
Obsessive Compulsive Disorder	52	14,253	9.7 (7.9–11.9)	92	633.62 (*p* < 0.0001)	(2, 9, 11, 14–17, 19, 22, 32, 34, 38, 41, 42, 46, 47, 57, 76, 83–85, 89–92, 95–100, 106, 107, 112, 115–117, 119, 120, 124, 125, 127–132, 134–136)
Social Anxiety Disorder	51	14,806	13.5 (11.3–16)	93.34	751 (*p* < 0.0001)	(2, 9, 11, 15–17, 19, 22, 34, 38, 42, 46, 47, 52, 57, 76, 77, 83, 84, 89–91, 95–97, 99, 100, 102, 103, 106, 107, 109, 115–117, 119, 120, 124, 125, 127–132, 134–139)
Generalized Anxiety Disorder	42	10,285	13.3 (10.7–16.5)	94.8	801.82 (*p* < 0.0001)	(11, 15–17, 19, 22, 25, 26, 32, 34, 38, 42, 46, 47, 52, 76, 77, 84, 89–91, 95–99, 102, 103, 106, 107, 109, 115, 116, 119, 123, 124, 128, 129, 132, 134, 136, 137)

### Risk of bias and quality assessment

Publication bias assessment by inspection of the funnel plot revealed symmetrical distribution of the included studies. Egger's regression test [intercept: −1.29 (−4.7, 2.1), *p*-value: 0.44] and Kendall's tau with continuity correction test (tau: −0.0108, *p*-value: 0.92) also did not indicate a publication bias when data from studies on BD and any anxiety disorder comorbidity are analyzed.

Heterogeneity of the studies were calculated for each analysis and are presented in Tables 1, 2. For each analysis, included studies showed significant heterogeneity. All subgroup analysis revealed at least moderate heterogeneity of the studies (I2 > 80%) and most subgroup analysis revealed high heterogeneity (I2 > 90%) of the included studies (Tables 1, 2).

**Table 2:** Current comorbidity of anxiety disorders in bipolar disorder.

**Current Comorbidity**	**No of studies**	**No of Patients**	**Prevalence % (CI)**	**Heterogeneity I2**	**Cochran Q (*p*-value)**	**References**
Any anxiety disorder	30	10,590	38.2 (32.8–43.9)	96.1	742.35 (*p* < 0.0001)	(6, 10, 16, 17, 19, 21, 42, 49, 54, 76, 90, 92, 93, 97, 107, 114, 122, 140–152)
Panic disorder	34	7,394	13.3 (10.8–16.3)	90.9	363.9 (*p* < 0.0001)	(10, 16, 19, 21, 28, 31, 42, 43, 49, 54, 62, 76, 90, 92, 93, 107, 114, 122, 126, 140, 142–145, 147, 149, 152–159)
Obsessive compulsive disorder	32	7,134	9.9 (7.9–12.4)	87.9	258.2 (*p* < 0.0001)	(10, 16, 19, 21, 28, 41, 42, 49, 54, 62, 76, 84, 90, 92, 93, 107, 114, 122, 126, 140, 142–145, 147, 152–154, 156–158, 160)
Social anxiety disorder	23	5,361	11.7 (8.6–15.8)	93.3	326.8 (*p* < 0.0001)	(10, 16, 19, 21, 28, 42, 49, 54, 76, 90, 93, 107, 122, 126, 140, 142, 143, 145, 147, 152, 154, 155, 158)
Generalized anxiety disorder	28	6,529	15.2 (11–20)	95.4	590.78 (*p* < 0.0001)	(10, 16, 19, 21, 28, 31, 42, 49, 54, 62, 76, 90, 93, 107, 122, 126, 127, 140, 142, 145, 147, 152–154, 156–159)

### Prevalence of anxiety disorders in bipolar disorder

We found that any DSM lifetime anxiety disorder comorbidity in BD is 40.5%; panic disorder comorbidity is at 18.1%, generalized anxiety disorder 13.3%, social anxiety disorder 13.5% and obsessive compulsive disorder 9.7% (Table 1). Any DSM current anxiety disorder comorbidity in BD is 38.2%; generalized anxiety disorder comorbidity is at 15.2%, panic disorder 13.3%, social anxiety disorder 11.7% and obsessive compulsive disorder 9.9% (Table 2).

**Figure 2:**
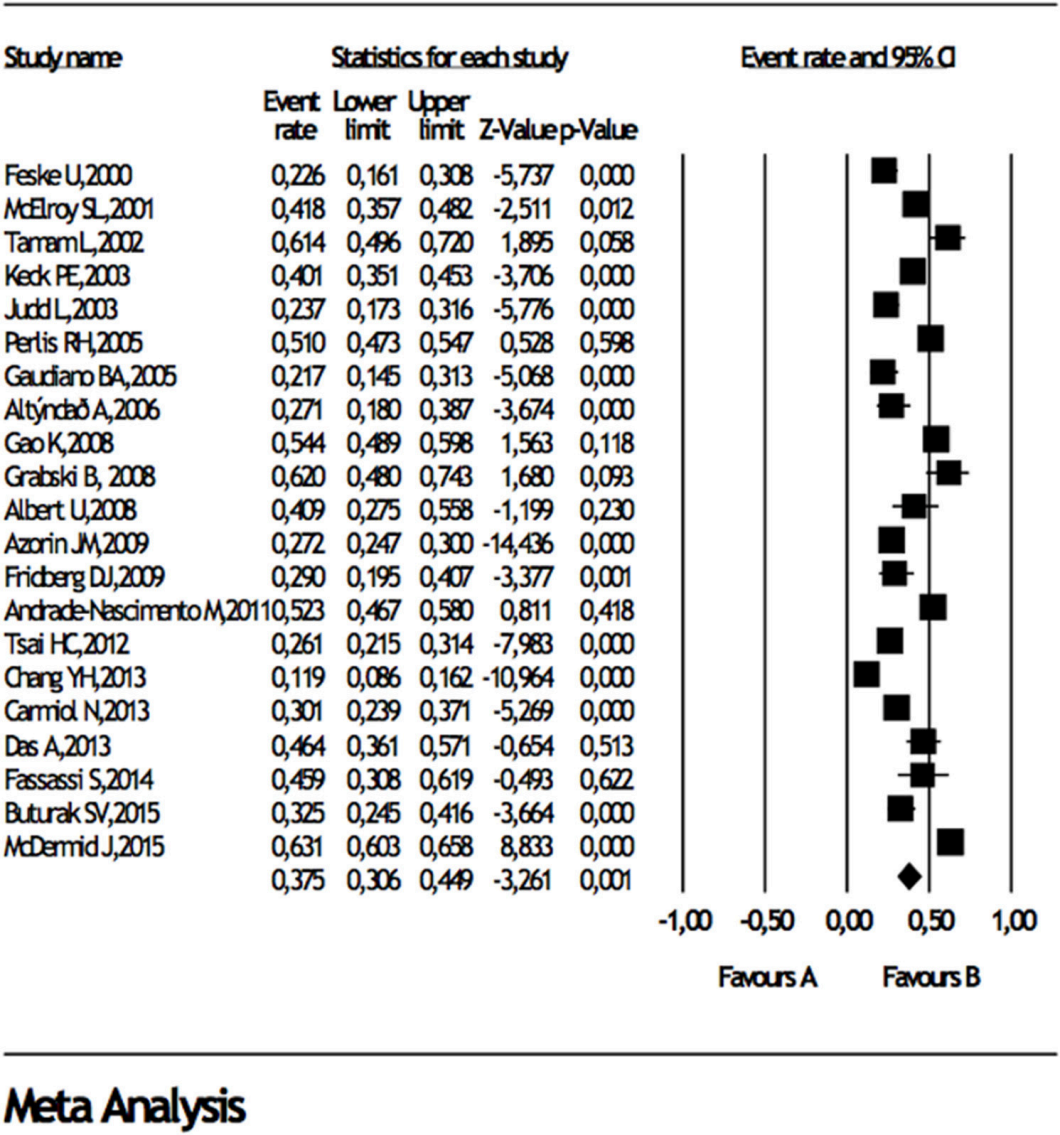
Forest plot for lifetime comorbidity of any anxiety disorder diagnosis in bipolar disorder I.

**Figure 3:**
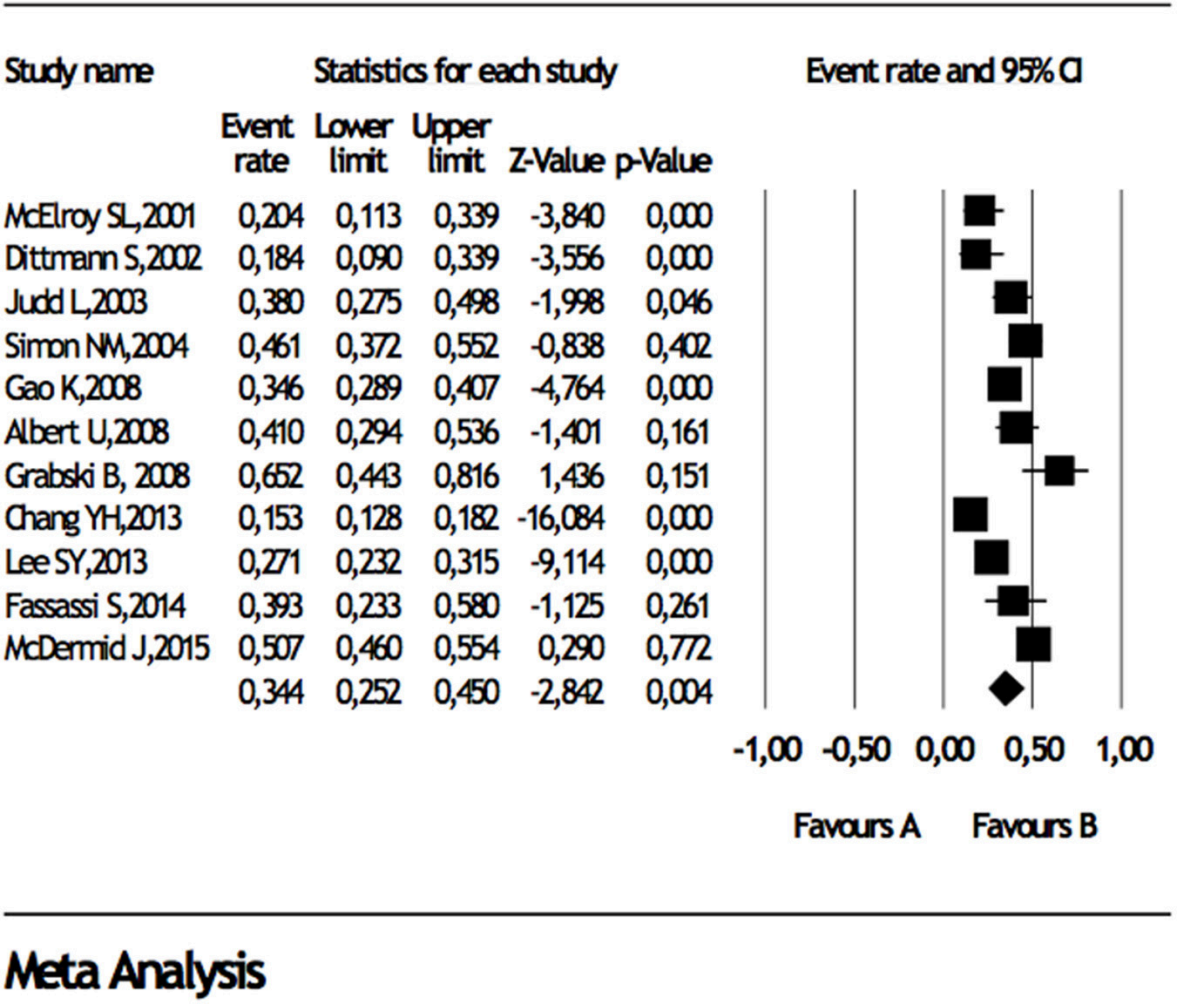
Forest plot for lifetime comorbidity of any anxiety disorder diagnosis in bipolar disorder II.

When studies reporting data about comorbidities in BD I or BD II were analyzed, separately, any DSM lifetime anxiety disorder comorbidity in BD I and BD II were 38 and 34%, panic disorder was 15 and 15%, generalized anxiety disorder was 14 and 16.6%, social anxiety disorder was 8 and 13%, obsessive compulsive disorder was 8% and 10%, respectively (Table 3), Forest plots for lifetime any anxiety disorder diagnosis in BD I and BD II are presented in Figure 2 and Figure 3, respectively. Any DSM current anxiety disorder comorbidity in BD I or BD II were 31 and 37%, panic disorder was 9 and 13%, generalized anxiety disorder was 8 and 12%, social anxiety disorder was 7 and 11%, and obsessive compulsive disorder was 8 and 7%, respectively (Table 4).

**Table 3:** Lifetime comorbidity of anxiety disorders in bipolar disorder I and bipolar disorder II.

**Lifetime Comorbidity**	**BD I**				**BD II**			
	**No of studies**	**No of Patients**	**Prevalence % (CI)**	**References**	**No of studies**	**No of patients**	**Prevalence % (CI)**	**References**
Any anxiety disorder	21	5,842	38 (31–45)	(13, 16, 17, 34, 42, 46, 51, 56, 76, 87, 90, 95, 98, 99, 101, 103, 105, 106, 109, 110, 161)	11	2,171	34 (25–45)	(13, 27, 42, 55, 90, 98, 99, 105, 106, 109, 162)
Panic disorder	26	5,831	15 (11–19)	(9, 11, 13, 16–18, 34, 42, 46, 52, 76, 84, 90, 95, 98, 99, 103, 106, 109, 112, 121, 125, 129, 134, 161, 163)	15	1,823	15 (11–20)	(9, 11, 13, 27, 42, 52, 90, 98, 99, 106, 109, 121, 129, 136, 163)
Obsessive compulsive disorder	21	4,133	8 (6–12)	(9, 11, 13, 16, 17, 34, 42, 46, 76, 84, 90, 95, 98, 99, 106, 112, 125, 129, 134, 161, 163)	12	1,320	10 (7–14)	(9, 11, 13, 27, 42, 90, 98, 99, 106, 129, 136, 163)
Social anxiety disorder	22	5,335	8 (6–12)	(9, 11, 13, 16, 17, 34, 42, 46, 52, 76, 84, 90, 95, 99, 103, 106, 109, 125, 129, 134, 161, 163)	13	1,526	13 (9–16)	(9, 11, 13, 27, 42, 52, 90, 99, 106, 109, 129, 136, 163)
Generalized anxiety disorder	20	4,968	14 (11–19)	(11, 13, 16, 17, 34, 42, 46, 52, 76, 84, 90, 95, 98, 99, 103, 106, 109, 129, 134, 161)	12	1,516	16.6 (14–20)	(11, 13, 27, 42, 52, 90, 98, 99, 106, 109, 129, 136)

**Table 4:** Current comorbidity of anxiety disorders in bipolar disorder I and bipolar disorder II.

**Current Comorbidity**	**BD I**				**BD II**			
	**No of studies**	**No of Patients**	**Prevalence % (CI)**	**References**	**No of studies**	**No of Patients**	**Prevalence % (CI)**	**References**
Any anxiety disorder	14	2,590	31 (24–39)	(16, 17, 21, 27, 29, 42, 76, 90, 93, 114, 143, 144, 150, 152)	7	730	37 (26–48)	(27, 42, 90, 93, 114, 150, 152)
Panic disorder	13	2,315	9 (6–14)	(16, 21, 27, 42, 76, 90, 93, 114, 143, 144, 152, 155, 158)	6	651	13 (8–23)	(27, 42, 90, 93, 114, 152)
Obsessive compulsive disorder	14	2,545	8 (5–13)	(16, 21, 27, 42, 76, 84, 90, 93, 114, 143, 144, 152, 153, 158)	7	685	7 (3–15)	(27, 42, 90, 93, 114, 152, 153)
Social anxiety disorder	10	2,153	7 (4–11)	(16, 27, 42, 76, 90, 93, 143, 152, 155, 158)	5	544	11 (6–17)	(27, 42, 90, 93, 152)
Generalized anxiety disorder	9	1,968	8 (4–14)	(16, 21, 27, 42, 76, 90, 93, 152, 158)	5	544	12 (9–16)	(27, 42, 90, 93, 152)

### Meta-regression analysis

We conducted a univariate analysis using mixed effect regression (unrestricted maximum likelihood) analysis to explore the source of heterogeneity. We included many variables that may influence anxiety disorder - BD comorbidity and found that the percentage of patients in a manic episode in the sample significantly affected heterogeneity of the any anxiety disorder comorbidity in BD. This was true for both lifetime and current comorbidity (Z: −2.14 and Z: −2.02, *p* = 0.03 and *p* = 0.04, respectively) and there was also a trend for an inverse correlation for other lifetime anxiety disorders and significant negative correlations with current anxiety disorders as well. References for the studies used in meta-regression analysis of lifetime comorbidity are presented in supplementary Table 1 and references for the studies used in meta-regression analysis of current comorbidity are presented in supplementary Table 2.

The results of the univariate analysis for each covariate and anxiety disorder - BD lifetime and current comorbidity can be found in Tables 5, 6. Age of onset of BD tended to have a significant or near significant impact on other anxiety disorders as well (Z: −1.77, *p* = 0.076 for lifetime any anxiety disorder comorbidity, Z: −1.75, *p* = 0.08 for lifetime panic disorder comorbidity, Z: −1.52, *p* = 0.13 for lifetime panic disorder comorbidity, Z: −2.72, *p* = 0.006 for lifetime social anxiety disorder comorbidity, Z: −1.93, *p* = 0.053 for current any anxiety disorder, Z: −3.6, *p* = 0.0003 for current panic disorder comorbidity, Z: −1.73, *p* = 0.08 for current OCD comorbidity, Z: −3.85, *p* = 0.0001 for current SAD comorbidity). In addition, percentage of BD I patients significantly affected heterogeneity of the studies by decreasing the prevalence of panic disorder and social anxiety disorder (Z: −2.2, *p* = 0.026 and *Z* = −2.34, *p* = 0.019). Mean duration of illness and mean age of the study populations affected lifetime obsessive compulsive disorder comorbidity inversely (Z: −2.2, *p* = 0.02 and Z: −2.4, *p* = 0.01). However, mean duration of BD significantly predicted current panic disorder comorbidity (Z: 2.96, *p* = 0.003).% of patients with an ADHD diagnosis significantly predicted lifetime comorbidity of panic disorder (Z: 3.27 *p* = 0.001) and both lifetime and current OCD (Z: 3.17, *p* = 0.001, Z: 4.7, *p* < 0.00001). Here we note two of the stronger and intriguing findings from this table: first, the percentage of patients with a substance use disorder affected comorbidity of both current and lifetime social anxiety disorder (Z: 2.1, *p* = 0.03 and Z: 2.27, *p* = 0.02). In other words, a higher rate of substance use disorder was associated with greater BD—social anxiety disorder. Second, a history of psychotic features significantly affected current (but not lifetime) panic disorder and GAD (Z: −4.22, *p* = 0.00003 and Z: −2.94, *p* = 0.0003, respectively). In other words, BD patients with psychotic features tended to report lower rates of current BD—panic and generalized anxiety disorder comorbidity.

**Table 5:** Analysis of possible associated features that may affect the heterogeneity of lifetime anxiety disorder comorbidity in bipolar disorder.

	**Lifetime Comorbidity**									
	**Any anxiety disorder**		**Panic disorder**		**Obsessive compulsive disorder**		**Social anxiety disorder**		**Generalized anxiety disorder**	
	**No of Studies**	***p*****-value**	**No of Studies**	***p*****-value**	**No of Studies**	***p*****-value**	**No of Studies**	***p*****-value**	**No of Studies**	***p*****-value**
% of BD I patients	35	0.43	49	0.99	41	0.31	40	0.33	33	0.85
Mean Age	38	0.87	55	0.82	48	**0.01**	46	0.98	35	0.49
% of Married patients	17	0.07	23	0.25	18	0.60	22	0.52	17	0.65
% of patients in a manic episode	12	**0.03**	19	0.08	18	**0.006**	19	**0.02**	16	0.80
% with a history of psychosis	12	0.44	22	0.92	20	0.95	21	0.31	14	0.34
% of high school and higher graduates	7	0.28	10	0.22	8	0.28	9	0.30	8	0.17
% of females	37	0.91	54	0.07	46	0.48	45	0.87	35	0.38
Duration of illness	14	0.20	12	0.83	11	**0.02**	13	0.24	11	0.91
Age of onset	30	0.07	46	0.08	38	0.13	38	**0.006**	31	0.52
% of patients with ADHD			4	**0.001**	3	**0.001**	3	**0.00002**	3	0.9
% of patients with SUD	30	0.15	46	0.26	38	0.44	35	**0.02**	28	0.31
Family history of BD	7	0.1	10	**0.05**	9	0.74	9	0.56	6	0.72

*The bold values indicate significant variables*.

**Table 6:** Analysis of possible associated features that may affect the heterogeneity of current anxiety disorder comorbidity in bipolar disorder.

	**Current comorbidity**									
	**Any anxiety disorder**		**Panic disorder**		**Obsessive compulsive disorder**		**Social anxiety disorder**		**Generalized anxiety disorder**	
	**No of studies**	***p*-value**	**No of studies**	***p*-value**	**No of studies**	***p*-value**	**No of studies**	***p*-value**	**No of studies**	***p*-value**
% of BD I patients	24	0.16	26	**0.026**	26	0.23	18	**0.019**	22	0.098
Mean Age	26	0.81	29	0.56	27	0.16	19	0.82	23	0.24
% of Married patients	13	0.63	12	0.41	12	0.84	9	0.64	11	0.25
% of patients in a manic episode	15	**0.04**	19	**0.04**	17	0.16	12	0.10	15	**0.00005**
% with a history of psychosis	6	0.61	9	**0.00003**	6	0.98	7	0.11	9	**0.0003**
% of high school and higher graduates	5	0.50	8	**0.017**	7	0.56	6	0.19	8	0.19
% of females	28	0.47	31	0.07	29	0.86	20	0.62	26	**0.03**
Duration of illness	8	0.53	10	**0.003**	9	0.52	9	0.45	10	0.09
Age of onset	20	**0.05**	22	**0.0003**	23	0.08	18	**0.00011**	19	0.16
% of patients with ADHD			4	0.90	4	**0.0000**	3	**0.0000**	4	0.35
% of patients with SUD	23	**0.05**	25	**0.04**	24	0.96	17	**0.03**	22	0.43

*The bold values indicate significant variables*.

## Discussion

### Design of the study

To our knowledge, this study is the most comprehensive and selective meta-analysis of anxiety disorder comorbidity in BD. Several design features distinguish our study from two recently published meta-analyses on a similar topic (65, 66), as follows. We accessed a very large number of published articles in this work and we examined studies with overlapping samples so as to include as much as unique data as possible. We discriminated reported current and lifetime prevalence of anxiety disorders in our analyses. We did not include studies that reported additive prevalence of subsets of anxiety disorders as “any anxiety disorder.” Also, though we noted these variables separately from all studies and used BD I proportion in the sample as a covariate, in case a study reported prevalences separately for BD I and other BD spectrums, we summed them all to model all BD group. Therefore, all BD group in this study does not stand for BD I alone as in Nabavi et al. (66). We also chose to define the current status of the patients as the percentage of patients in a manic episode, which distinguished our findings from the previous studies as Preti et al. (67) and Pavlova et al. (68). We believe these features provide a comprehensive look at the literature on this topic so far.

### Prevalence of anxiety disorders in BD

We found that both lifetime and current anxiety disorder diagnosis are highly prevalent in BD patients. Generalized anxiety disorder and panic disorder are the most prevalent diagnoses, followed by social anxiety disorder and obsessive compulsive disorder. Our findings are in accordance with Nabavi et al. (66), but we found lower rates for anxiety disorders other than OCD, when compared to Pavlova et al. (65). Methodological differences, number of studies included in the analysis and data extraction methods are likely to be responsible for this variance. We also found that all anxiety disorder comorbidities are much higher than expected for the general population and even for disorders as schizophrenia (69, 70). This analysis continues to support a close association between bipolar disorder and anxiety disorders, above and beyond the usual associations seen between different kinds of psychopathology. There are likely to be shared environmental and neurobiological pathways for the co-occurrence of these two conditions. An anxiety disorder diagnosis may precede the diagnosis of BD, essentially as a prodromal feature (19). As environmental factors, early life adversities (71–73) and social rhytm disruptions (74) may all play a role for in both disorders and lead to increased HPA axis activity, which changes the excitability of the cortex and activation of relevant networks. In addition, amygdala, which is a core tuning center for both fear and reward networks that regulates emotion and anxiety is hyperactive in both disorders (74) which might predispose individuals to both anxiety symptoms and mood instability. Lastly, both disorders are associated with decreased executive functioning and prefrontal cortex activity, which may predispose to negative schema activation and decreased control and impaired perception over environmental concepts that includes both fear and social clues (74).

### Lifetime and current anxiety-BD comorbidity

Because of the methodology and reported data categories of the included studies, studies included in the lifetime comorbidity analysis did not overlap with studies included in the current comorbidity analysis. Still, we found very consistent findings showing higher lifetime comorbidity compared to current comorbidity except for current GAD prevalance which was found to be higher than lifetime GAD comorbidity. Heterogeneity, sample size and used structural clinical interviews may have lead to this finding.

### Comparison of BD I and BD II

In our meta-analysis, we found higher prevalence of anxiety disorders in BD II compared to BD I, except for lifetime any anxiety disorder diagnosis and a current OCD diagnosis. The increased lifetime prevalence of anxiety disorders in BD I may be due to higher rates of specific phobia or PTSD comorbidity in BD I which we did not analyze. There may also be a higher number of heterogeneous studies leading to this outcome for BD I. Our findings for anxiety disorder comorbidity in BD I vs. BD II are different from those of Pavlova et al. (65). In that paper, investigator pooled studies that concurrently include data for BD I and BD II to calculate a risk ratio for anxiety disorders. Here, we presented a prevalence analysis instead of a risk ratio calculation. We also conducted a meta-regression analysis to study the effect of BD I percentage in the sample on all studies for this purpose. This analysis can be thought as comparing BD I percentage to all other BD spectrum disorders. In this analysis, the proportion of BD I in the sample did not have a significant influence on the heterogeneity of lifetime comorbidity, but it did affect current panic and social anxiety disorder comorbidity. This finding indicates that the close relationship between BD and anxiety disorders is indeed seen across the bipolar spectrum with some modest differences between BD I and BD II. Nonetheless, the elevated rates of anxiety disorder comorbidity in BD II may be responsible for increased suicidality in this patient group (52).

### Associated features of anxiety disorder comorbidity

Among the sociodemographic variables that we could included in the analysis, marital status of the patients did not influence either lifetime or current anxiety disorder comorbidity and educational level explained the heterogeneity for only current PD. Patients with a high school or higher education reported higher current panic disorder. For these two variables, we had dichotomized the variables to two groups while extracting the data from the articles for homogeneity, however this design may have limited our observation for a significant difference for the effect of these variables.

We found that age of onset is a significant covariate for anxiety disorder comorbidity. In our analysis, we took into account the mean age of onset for the sample. In a recent meta-analyses by Joslyn et al, data from 10 studies about the impact of early and adult onset of BD on severity measures of BD was examined and a 1.72-fold elevated anxiety disorder comorbidity risk was seen with earlier BD onset (75). The rest of the literature also supports the observation that early onset BD patients may be at higher risk of an anxiety disorder diagnosis (19, 30, 76, 77). Based on our findings, age of onset of bipolar disorder negatively correlates with almost all anxiety disorders. This finding may support the discussion that bipolar disorder patients may have subgroups based on the age of onset, where prevalance of anxiety disorders are higher in the earlier onsets, but may decrease with later onsets. Medications used for treatment and neurobiological changes related with age may lead to this finding. On the other hand, duration of illness mainly did not affect AD-BD comorbidity except for negatively correlation with lifetime OCD and positively correlation with current PD. Mean age of the sample was also not significant mostly. When findings about age of onset, duration of illness and mean age of the sample is combined, it can be concluded that the age of onset is a more significant determinant of anxiety disorder comorbidity compared to age of the participant and duration of illness.

The finding of elevated rates of anxiety disorder comorbidity in samples with higher proportion of patients in manic episode is intriguing. Percentage of patients in a manic episode significantly explained the heterogeneity of almost all lifetime and current anxiety disorders' comorbidity. Similarly, the proportion of patients with psychotic features affected rates of current (but not lifetime) panic and generalized anxiety disorder diagnoses. A history of psychosis is known to impart a worse outcome for BD (78) but it is not clear what the implication would be for a current anxiety disorder diagnosis. Patients experiencing mania or psychosis may be less likely to endorse anxiety symptoms, or to report them. We are not aware of a literature questioning the background of this finding.

Also, our findings highlight specific relationship between substance use disorders and social anxiety disorder comorbidity in BD. This relationship is reminiscent of the extensive literature on social anxiety and substance use disorder (79–81), and it is reassuring to observe it in our analysis. Lastly, even though the number of studies included in the analysis were very low because of the current published literature, ADHD comorbidity significantly increased reported anxiety disorders comorbidity. As discussed in the second discussion section, ADHD also shares common neurobiological pathways with anxiety and mood disorders on dysfunction of executive control and emotion regulation, studies assessing the ADHD-anxiety-mood disorders epidemiological significance and neurobiological overlaps are needed to clarify better treatment approaches to both disorders, as well.

### Limitations of the study and current literature

Our study has some limitations. Most studies in our analysis are not population-based but rather recruited patients from outpatient or inpatient clinical services. In this context, they did not report a nonresponse rate, which reduces generalizability. Even though we tried to model clinical remission through the percentage of inpatients in the sample, our results may not be representative for intra-episode comorbidity. Most of the studies did not define what they meant by “current” disorder diagnosis except for Gao et al. (82). Studies mainly provided knowledge about mean age of onset (as opposed to a distribution), and this may not be reliable for most of the studies. Indeed, the age of onset definition varied across studies [e.g., (22, 83, 84)], define age of onset as the time point where the patient has enough symptoms to meet the diagnostic criteria of BD, whereas Edmonds et al. (85) defines as age at first professional contact, Goldstein and Levitt (86) defines as age of mania onset and Keck et al. (87) defines as age of first treatment. We did not have detailed data about educational levels in the samples, and therefore decided to generate a categorical variable of “high school or higher education.” Family history of anxiety disorder in the sample was not reported in most studies; we therefore could not include these variables in the regression analysis. Our meta-regression analysis is mainly a univariate analysis, which does not analyse the interaction of the various variables we assessed. Interaction of ADHD and SUD diagnosis on anxiety comorbidity and mood episodes of BD would be important to focus in new population studies.

## Conclusion and future perspectives

The main findings of our study can be concluded as follows: 1: Anxiety disorder comorbidity is high in BD with somewhat lower rates in BD I vs. BD II, 2: Proportion of patients in a manic episode or with psychotic features influences anxiety disorder comorbidity, which may also explain lower rates in BD I, 3: Patients with an earlier age of BD onset report higher anxiety disorder comorbidity and age of onset for BD is a more important factor compared to duration of illness and current age for determining AD comorbidity, 4: Social anxiety comorbidity in BD is associated with substance use disorders, and 5: ADHD diagnosis in the population significantly influences anxiety disorder comorbidity.

Further research may analyze how current depressive and manic scores or suicidal ideation may change the diagnosis of current comorbidity of anxiety disorders and how the comorbidity of anxiety disorders may affect the treatment response. The effect of previous number of bipolar disorder episodes (both for manic, depressive or mixed states) or rapid cycling features, number of previous hospitalizations, lifetime suicide attempts, and association of anxiety disorder diagnosis with other comorbid medical diagnosis should also be studied. Anxiety disorders may also be related to different cognitive phenotypes when BD I and BD II are compared (44), role of anxiety disorders in clustering and subtyping of bipolar spectrum may be studied further. On the other hand, focusing on the neurobiology and shared environmental factors associated with AD and BD may pave the way for better future treatments. Finally, we need studies for the effect of medications on anxiety disorders comorbidity as seen in OCD-schizophrenia comorbidity (70, 88).

## Author contributions

HY and DO have designed the study. HY, AK, CK, and MY have conducted the literature search and exclusion of the data. HY and AK conducted the statistical analysis. All authors contributed significantly to the discussion of the findings. HY, AK and DO wrote the manuscript. All authors approved the final version of the article.

### Conflict of interest statement

DO was on a Scientific Advisory Board for Neurocrine Inc. in 2016. The remaining authors declare that the research was conducted in the absence of any commercial or financial relationships that could be construed as a potential conflict of interest.
