# Supplementary material for: Prevalence and Associated Features of Anxiety Disorder Comorbidity in Bipolar Disorder: A Meta-Analysis and Meta-Regression Study
Source: Front Psychiatry. 2018 Jun 27;9:229. doi: 10.3389/fpsyt.2018.00229 (PMC6030835; doi:10.3389/fpsyt.2018.00229)
Supplement: Supplementary file 1 [file Data_Sheet_1.docx]

Supplementary Table 1:List of references used in the analysis of Possible Associated Features that may affect the heterogeneity of Lifetime Anxiety Disorder Comorbidity in Bipolar Disorder

| **% of BD I patients** | Any Anxiety Disorder | (13, 16, 17, 19, 22, 30, 39, 40, 42, 46, 51, 55, 56, 76, 89, 90-93, 95-110) |
| --- | --- | --- |
|  | Panic disorder | (9, 11, 15-19, 22, 30, 38, 42, 46, 52, 57, 76, 77, 83-85, 89-92, 95-100, 102, 103, 106, 107, 109, 112, 114, 118, 119, 121, 124-129, 131, 134-136) |
|  | Obsessive Compulsive Disorder | (9, 11, 15-17, 19, 22, 38, 41, 42, 46, 48, 57, 76, 77, 83-85, 89-92, 95-100, 106, 107, 112, 114, 119, 124, 125, 127-129, 131, 134-136) |
|  | Social Phobia | (9, 11, 15-17, 19, 22, 38, 42, 46, 52, 57, 76, 77, 83, 84, 89, 90, 91, 95-97, 99, 100, 102, 103, 106, 107, 109, 119, 124, 125, 127-129, 131, 134-136, 139) |
|  | Generalized Anxiety Disorder | (11, 15-17, 19, 22, 25, 38, 42, 46, 52, 76, 77, 84, 89, 90, 91, 95-99, 102, 103, 106, 107, 109, 119, 124, 128, 129, 134, 136) |
| **Mean Age** | Any Anxiety Disorder | (13, 16, 17, 19, 22, 30, 39, 40, 42, 45-48, 51, 55, 76, 82, 89-92, 94-108, 110, 111) |
|  | Panic disorder | (2, 9, 11, 15-19, 22, 30, 32, 38, 42, 46-47, 57, 76, 77, 83-86, 89-92, 95-100, 102, 103, 106, 107, 112, 114, 116-121, 124-131, 134-136) |
|  | Obsessive Compulsive Disorder | (2, 9, 11, 15-17, 19, 22, 32, 38, 41, 42, 46-47, 57, 76, 77, 83-85, 89-92, 95-100, 106, 107, 112, 114, 116, 117, 119, 120, 124, 125, 127-131, 134-136) |
|  | Social Phobia | (2, 9, 11, 15-17, 19, 22, 38, 42, 46-47, 57, 76, 77, 83, 84, 86, 89-91, 95-97, 99, 100, 102, 103, 106, 107, 116, 117, 119, 120, 124, 125, 127-131, 134-136, 138, 139) |
|  | Generalized Anxiety Disorder | (11, 15-17, 19, 22, 25, 32, 38, 42, 46-47, 76, 77, 84, 86, 89-91, 95-99, 102, 103, 106, 107, 116, 119, 124, 128, 129, 134, 136) |
| **% of Married patients** | Any Anxiety Disorder | (13, 16, 17, 19, 45, 46, 48, 76, 89, 99, 100, 102, 103, 107, 108, 110, 165) |
|  | Panic disorder | (16, 17, 19, 36, 46, 48, 76, 86, 89, 99, 100, 102, 103, 107, 113, 116, 121, 125, 126, 129, 130, 134, 136) |
|  | Obsessive Compulsive Disorder | (16, 17, 19, 36, 41, 46, 48, 76, 89, 99, 100, 107, 116, 125, 129, 130, 134, 136) |
|  | Social Phobia | (16, 17, 19, 46, 48, 76, 86, 89, 99, 100, 102, 103, 107, 116, 125, 129, 130, 134, 136-139) |
|  | Generalized Anxiety Disorder | (16, 17, 19, 46, 48, 76, 86, 89, 99, 102, 103, 107, 116, 129, 134, 136, 137) |
| **% of patients in a manic episode** | Any Anxiety Disorder | (16, 17, 19, 42, 44, 46, 47, 76, 95, 97, 102, 104) |
|  | Panic disorder | (16, 17, 19, 42, 44, 46, 47, 76, 95, 97, 102, 112, 118, 121, 124, 125, 128, 129, 133, 134, 136) |
|  | Obsessive Compulsive Disorder | (16, 17, 19, 41, 42, 44, 46, 47, 76, 95, 97, 112, 124, 125, 128, 129, 134, 136) |
|  | Social Phobia | (16, 17, 19, 42, 44, 46, 47, 76, 95, 97, 102, 124, 125, 128, 129, 134, 136, 138, 139) |
|  | Generalized Anxiety Disorder | (16, 17, 19, 42, 44, 46, 47, 76, 95, 97, 102, 124, 128, 129, 134, 136) |
| **% with a history of psychosis** | Any Anxiety Disorder | (13, 16, 22, 44, 51, 76, 89, 91, 93, 95, 100, 107) |
|  | Panic disorder | (2, 16, 18, 22, 38, 44, 76, 83, 89, 91, 95, 100, 107, 115-119, 129, 131, 135, 136) |
|  | Obsessive Compulsive Disorder | (2, 16, 22, 38, 44, 76, 83, 89, 91, 95, 100, 107, 115-117, 119, 129, 131, 135, 136) |
|  | Social Phobia | (2, 16, 22, 38, 44, 76, 83, 89, 91, 95, 100, 107, 115-117, 119, 129, 131, 135, 136, 138) |
|  | Generalized Anxiety Disorder | (16, 22, 38, 44, 76, 89, 91, 95, 107, 115, 116, 119, 129, 136) |
| **% of high school and higher graduates** | Any Anxiety Disorder | (16, 19, 27, 76, 99, 108, 162) |
|  | Panic disorder | (16, 19, 27, 36, 76, 99, 121, 126, 129, 136) |
|  | Obsessive Compulsive Disorder | (16, 19, 27, 36, 76, 99, 129, 136) |
|  | Social Phobia | (16, 19, 27, 76, 99, 129, 136, 137, 139) |
|  | Generalized Anxiety Disorder | (16, 19, 27, 76, 99, 129, 136, 137) |
| **% of females** | Any Anxiety Disorder | (13, 16, 17, 19, 22, 30, 39, 40, 42, 46,47, 51, 55, 56, 76, 89-108, 110, 111) |
|  | Panic disorder | (2, 9, 11, 15-19, 22, 30, 32, 38, 42, 46, 47, 57, 76, 84-86, 90-92, 95-100, 102, 103, 106, 107, 112-114, 116-121, 124-131, 134-136, 163) |
|  | Obsessive Compulsive Disorder | (2, 9, 11, 15-17, 19, 22, 32, 38, 41, 42, 46, 47, 57, 76, 84, 85, 89-92, 95-100, 106, 107, 112, 114, 116, 117, 119, 120, 124, 125, 127-131, 134-136) |
|  | Social Phobia | (2, 9, 11, 15-17, 19, 22, 38, 42, 46,47, 57, 76, 84, 86, 89-91, 95-97, 99, 100, 102, 103, 106, 107, 116, 117, 119, 120, 124, 125, 127-131, 134-139) |
|  | Generalized Anxiety Disorder | (11, 15-17, 19, 22, 25, 32, 38, 42, 46, 47, 76, 84, 86, 89-91, 95-99, 102, 103, 106, 107, 116, 119, 124, 128, 129, 134, 136, 137) |
| **Duration of illness** | Any Anxiety Disorder | (19, 42, 44-47, 76, 91, 101, 104, 107, 108, 110, 166) |
|  | Panic disorder | (19, 38, 42, 44, 46, 47, 57, 76, 86, 91, 107, 129) |
|  | Obsessive Compulsive Disorder | (19, 38, 42, 44, 46, 47, 57, 76, 91, 107, 129) |
|  | Social Phobia | (19, 38, 42, 44, 46, 47, 57, 76, 86, 91, 107, 129, 138) |
|  | Generalized Anxiety Disorder | (19, 38, 42, 44, 46, 47, 76, 86, 91, 107, 129) |
| **Age of onset** | Any Anxiety Disorder | (13, 16, 17, 19, 22, 39, 42, 44, 46, 47, 51, 76, 89-91, 93, 95-97, 99-101, 103, 104, 106-108, 110, 111, 166) |
|  | Panic disorder | (16-19, 22, 32, 42, 44, 46, 47, 57, 76, 77, 83-86, 89-91, 95-97, 99, 100, 103, 106, 107, 113, 114, 116-119, 121, 122, 124-126, 128, 129, 131, 133-136) |
|  | Obsessive Compulsive Disorder | (14, 16, 17, 19, 22, 32, 42, 44, 46, 47 57, 76, 77, 83-85, 89-91, 95-97, 99, 100, 106, 107, 114, 116, 117, 119, 124, 125, 128, 129, 131, 134-136) |
|  | Social Phobia | (16, 17, 19, 22, 42, 44, 46, 47, 57, 76, 77, 83, 84, 86, 89-91, 95-97, 99, 100, 103, 106, 107, 116, 117, 119, 124, 125, 128, 129, 131, 134-136, 138, 139) |
|  | Generalized Anxiety Disorder | (16, 17, 22, 25, 32, 42, 44, 46, 47, 76, 77, 84, 86, 89-91, 95-97, 99, 103, 106, 107, 116, 119, 124, 128, 129, 134, 136) |
| **% of patients with ADHD** | Panic disorder | (91, 106, 126, 136) |
|  | Obsessive Compulsive Disorder | (91, 106, 136) |
|  | Social Phobia | (91, 106, 136) |
|  | Generalized Anxiety Disorder | (91, 106, 136) |
| **% of patients with SUD** | Any Anxiety Disorder | (13, 16, 17, 22, 30, 39, 40, 47, 51, 55, 56, 76, 86, 89, 90, 92-94, 96-101, 103, 104, 106-108, 161) |
|  | Panic disorder | (2, 9, 16-18, 22, 30, 32, 38, 47, 57, 76, 83-86, 89, 90, 92, 96-100, 103, 106, 107, 112, 113, 116, 117, 119, 120, 122-124, 126-128, 130-135, 161) |
|  | Obsessive Compulsive Disorder | (2, 9, 16, 17, 22, 32, 38, 41, 47, 57, 76, 83-85, 89, 90, 92, 96-100, 106, 107, 112, 116, 117, 119, 120, 124, 127, 128, 130-132, 134, 135, 161) |
|  | Social Phobia | (2, 9, 16, 17, 22, 38, 47, 57, 76, 83-84, 86, 89, 90, 96, 97, 99, 100, 103, 106, 107, 116, 117, 119, 120, 124, 127, 128, 130-132, 134, 135, 137, 161) |
|  | Generalized Anxiety Disorder | (16, 17, 22, 25, 32, 38, 47, 76, 84, 86, 89, 90, 96-99, 103, 106, 107, 116, 119, 123, 124, 128, 132, 134, 137, 161) |
| **Family history of BD** | Any Anxiety Disorder | (39, 42, 45, 76, 100, 104, 161) |
|  | Panic disorder | (2, 42, 76, 100, 117, 124, 128, 133, 134, 161) |
|  | Obsessive Compulsive Disorder | (2, 42, 76, 100, 117, 124, 128, 134, 161) |
|  | Social Phobia | (2, 42, 76, 100, 117, 124, 128, 134, 161) |
|  | Generalized Anxiety Disorder | (42, 76, 124, 128, 134, 161) |

Supplementary Table 2: List of references used in the analysis of Possible Associated Features that may affect the heterogeneity of current Anxiety Disorder Comorbidity in Bipolar Disorder

| **% of BD I patients** | Any Anxiety Disorder | (6, 10, 16, 17, 19, 21, 42, 49, 76, 90, 92, 93, 97, 107, 114, 141-145, 148-150, 152) |
| --- | --- | --- |
|  | Panic disorder | (10, 16, 19, 21, 28, 31, 42, 49, 62, 76, 90, 92, 93, 107, 114, 126, 142-145, 149, 152, 153, 155, 156, 158) |
|  | Obsessive Compulsive Disorder | (10, 16, 19, 21, 28, 41, 42, 49, 62, 76, 84, 90, 92, 93, 107, 114, 126, 142-145, 152, 153, 156, 158, 160) |
|  | Social Phobia | (10, 16, 19, 21, 28, 42, 49, 76, 90, 93, 107, 126, 142, 143, 145, 152, 155, 158) |
|  | Generalized Anxiety Disorder | (10, 16, 19, 21, 26, 28, 31, 42, 49, 62, 76, 90, 93, 107, 126, 127, 142, 145, 152, 153, 156, 158) |
| **Mean Age** | Any Anxiety Disorder | (6, 10, 16, 17, 19, 21, 42, 49, 54, 76, 90, 92, 97, 107, 114, 140-143, 145-151) |
|  | Panic disorder | (10, 16, 19, 21, 28, 31, 42, 43, 48, 49, 54, 62, 76, 90, 92, 107, 114, 126, 140, 142, 143, 145, 147, 149, 153, 155, 157-159) |
|  | Obsessive Compulsive Disorder | (10, 16, 19, 21, 28, 41, 42, 49, 54, 62, 76, 82, 84, 90, 92, 107, 114, 126, 140, 142, 143, 145, 147, 153, 157, 158, 160) |
|  | Social Phobia | (10, 16, 19, 21, 28, 42, 49, 54, 76, 90, 107, 126, 140, 142, 143, 145, 147, 155, 158) |
|  | Generalized Anxiety Disorder | (10, 16, 19, 21, 28, 31, 42, 49, 54, 62, 76, 90, 107, 126, 127, 140, 142, 145, 147, 153, 157-159) |
| **% of Married patients** | Any Anxiety Disorder | (6, 10, 16, 17, 19, 76, 107, 143, 145, 146, 148, 150, 151) |
|  | Panic disorder | (10, 16, 19, 20, 31, 62, 76, 107, 126, 143, 145, 157) |
|  | Obsessive Compulsive Disorder | (10, 16, 19, 20, 41, 62, 76, 107, 126, 143, 145, 157) |
|  | Social Phobia | (10, 16, 19, 20, 76, 107, 126, 143, 145) |
|  | Generalized Anxiety Disorder | (10, 16, 19, 20, 31, 62, 76, 107, 126, 145, 157) |
| **% of patients in a manic episode** | Any Anxiety Disorder | (6, 10, 16, 17, 19, 26, 42, 76, 97, 140, 141, 143, 144, 150, 152) |
|  | Panic disorder | (10, 16, 19, 26, 28, 31, 42, 43, 62, 76, 140, 143, 144, 152, 153, 155, 157-159) |
|  | Obsessive Compulsive Disorder | (10, 16, 19, 26, 28, 41, 42, 62, 76, 140, 143, 144, 152, 153, 157, 158, 160) |
|  | Social Phobia | (10, 16, 19, 26, 28, 42, 76, 140, 143, 152, 155, 158) |
|  | Generalized Anxiety Disorder | (10, 16, 19, 26, 28, 31, 42, 62, 76, 140, 152, 153, 157-159) |
| **% with a history of psychosis** | Any Anxiety Disorder | (16, 76, 93, 107, 141, 152) |
|  | Panic disorder | (16, 31, 76, 93, 107, 152, 155, 158, 159) |
|  | Obsessive Compulsive Disorder | (16, 76, 93, 107, 152, 158) |
|  | Social Phobia | (16, 76, 93, 107, 152, 155, 158) |
|  | Generalized Anxiety Disorder | (16, 26, 31, 76, 93, 107, 152, 158, 159) |
| **% of high school and higher graduates** | Any Anxiety Disorder | (16, 19, 27, 76, 145) |
|  | Panic disorder | (16, 19, 27, 76, 126, 145, 157, 159) |
|  | Obsessive Compulsive Disorder | (16, 19, 27, 76, 126, 145, 157) |
|  | Social Phobia | (16, 19, 27, 76, 126, 145) |
|  | Generalized Anxiety Disorder | (16, 19, 27, 76, 126, 145, 157, 159) |
| **% of females** | Any Anxiety Disorder | (6, 10, 16, 17, 19, 21, 42, 54, 76, 90, 92, 93, 97, 107, 114, 140-152) |
|  | Panic disorder | (10, 16, 19, 21, 28, 31, 42, 43, 54, 62, 76, 82, 90, 92, 93, 107, 114, 126, 140, 142-145, 147, 149, 152, 153, 155-159) |
|  | Obsessive Compulsive Disorder | (10, 16, 19, 21, 28, 41, 42, 54, 62, 76, 84, 90, 92, 93, 107, 114, 126, 140, 142-145, 147, 152, 153, 156-158, 160) |
|  | Social Phobia | (10, 16, 19, 21, 28, 42, 48, 54, 76, 90, 93, 107, 126, 140, 142, 143, 145, 147, 152, 155, 158) |
|  | Generalized Anxiety Disorder | (10, 16, 19, 21, 26, 28, 31, 42, 54, 62, 76, 90, 93, 107, 126, 127, 140, 142, 145, 147, 152, 153, 156-159) |
| **Duration of illness** | Any Anxiety Disorder | (10, 19, 20, 42, 76, 107, 142, 147) |
|  | Panic disorder | (10, 19, 20, 42, 76, 107, 142, 147, 158, 159) |
|  | Obsessive Compulsive Disorder | (10, 19, 20, 42, 76, 107, 142, 147, 158) |
|  | Social Phobia | (10, 19, 20, 42, 76, 107, 142, 147, 158) |
|  | Generalized Anxiety Disorder | (10, 19, 20, 42, 76, 107, 142, 147, 158, 159) |
| **Age of onset** | Any Anxiety Disorder | (6, 10, 16, 17, 19, 21, 42, 54, 76, 90, 93, 97, 107, 114, 122, 141, 143, 145, 147, 164) |
|  | Panic disorder | (10, 16, 19, 21, 28, 42,54, 62, 76, 90, 93, 107, 114, 122, 126, 143, 145, 147, 153, 155, 157, 158) |
|  | Obsessive Compulsive Disorder | (10, 16, 19, 21, 28, 42, 54, 62, 76, 84, 90, 93, 107, 114, 122, 126, 143, 145, 147, 153, 157, 158, 160) |
|  | Social Phobia | (10, 16, 19, 21, 28, 42, 54, 76, 90, 93, 107, 122, 126, 143, 145, 147, 155, 158) |
|  | Generalized Anxiety Disorder | (10, 16, 19, 21, 28, 42, 54, 62, 76, 90, 93, 107, 122, 126, 145, 147, 153, 157, 158) |
| **% of patients with ADHD** | Panic disorder | (28, 126, 152, 157) |
|  | Obsessive Compulsive Disorder | (28, 126, 152, 157) |
|  | Social Phobia | (28, 126, 152) |
|  | Generalized Anxiety Disorder | (28, 126, 152, 157) |
| **% of patients with SUD** | Any Anxiety Disorder | (6, 10, 16, 17, 54, 76, 90, 92, 93, 97, 107, 122, 141, 143-150, 152, 164) |
|  | Panic disorder | (10, 16, 28, 31, 54, 62, 76, 90, 92, 93, 107, 122, 126, 143-145, 147, 149, 152-156, 158, 159) |
|  | Obsessive Compulsive Disorder | (10, 16, 28, 41, 54, 62, 76, 84, 90, 92, 93, 107, 122, 126, 143-145, 147, 152-154, 156, 158, 160) |
|  | Social Phobia | (10, 16, 26, 28, 54, 76, 90, 93, 107, 122, 143, 145, 147, 152, 154, 155, 158) |
|  | Generalized Anxiety Disorder | (10, 16, 26, 28, 31, 54, 62, 76, 90, 93, 107, 122, 126, 127, 145, 147, 152-154, 156, 158, 159) |
